# Validity of a Screening Tool for Patients with a Sub-Threshold Level of Lumbar Instability: A Cross-Sectional Study

**DOI:** 10.3390/ijerph182212151

**Published:** 2021-11-19

**Authors:** Arisa Leungbootnak, Rungthip Puntumetakul, Jaturat Kanpittaya, Thiwaphon Chatprem, Rose Boucaut

**Affiliations:** 1 Research Center of Back, Neck, Other Joint Pain and Human Performance (BNOJPH), Department of Physical Therapy, Faculty of Associated Medical Sciences, Khon Kaen University, Khon Kaen 40002, Thailand; rung.smd28@gmail.com (A.L.); thiwaphon.ao@gmail.com (T.C.); 2 Department of Radiology, Faculty of Medicine, Khon Kaen University, Khon Kaen 40002, Thailand; jatkan@gmail.com; 3 International Centre for Allied Health Evidence (iCAHE), School of Health Sciences (Physiotherapy), University of South Australia, Adelaide, SA 5001, Australia; rose.boucaut@unisa.edu.au

**Keywords:** sub-threshold lumbar instability, non-radiological lumbar instability, lumbar instability, radiography, lumbar translation, lumbar rotation, screening tool, X-ray, sensitivity, specificity

## Abstract

Lumbar instability (LI) comprises one subgroup of those with chronic low back pain (CLBP); it indicates the impairment of at least one of the spinal stabilizing systems, and radiographic criteria of translation and rotation are used for its diagnosis. Previous studies have developed and tested a screening tool for LI where patients with sub-threshold lumbar instability (STLI) were detected in the initial stage of lumbar pathology using radiographs as a gold standard for diagnosis. The radiographic measurement in STLI lies between the range of translation and rotation of the LI and asymptomatic lumbar motion. However, there are no studies indicating the validity and cut-off points of the screening tool for STLI. The current study aimed to determine the validity of an LI screening tool to support the diagnostic process in patients with STLI. This study design was cross-sectional in nature. A total of 135 participants with CLBP, aged between 20 and 60 years, who had undergone flexion and extension radiographs, answered a screening tool with 14 questions. The cut-off score for identifying STLI using the screening tool was at least 6/14 positive responses to the LI questions. The findings suggested that the LI screening tool we tested is effective for the detection of STLI. The tool can be used in outpatient settings.

## 1. Introduction

Lumbar instability (LI) is defined as excessive translation and rotation motion values of each lumbar segment compared with normal values [1], and dysfunction of one of the spinal stabilizing systems, which consist of three subsystems: passive, active, and neural [2,3]. LI is considered to be a subgroup of mechanical low back pain (MLBP) [4]. Previous articles have demonstrated that 12–57% [1,5,6,7,8,9] of patients with CLBP have lumbar instability. Patients with LI report a number of symptoms, including: pain [10], muscle spasms [11,12], abnormal movement quality [13], functional disability [5,10,12], and diminished quality of life [12]. The severity of LI can evolve and may require surgical stabilization [14].

LI can be measured using a flexion–extension radiograph as the gold standard for diagnosis. Patients with LI were defined by White and Panjabi (1990) using flexion–extension radiographic characteristics. Their criteria were: a sagittal plane translation greater than 4.5 mm, or sagittal plane rotation of greater than 15° at L1/L2, L2/L3, or L3/L4, greater than 20° at L4/L5, or greater than 25° at L5/S1 [15].

Staub and colleagues reported that, for asymptomatic volunteers, with an age range of 18 to 82 years, the value of sagittal translation at each lumbar segment was: 1.9 mm at L1/L2, 2.4 mm at L2/L3, 2.7 mm at L3/4, 2.8 mm at L4/L5, and 0.5 mm at L5/S1. The sagittal plane rotation ranges were 11.0° at L1/L2, 12.6° at L2/L3, 13.3° at L3/4, 14.7° at L4/L5, and 12.8° at L5/S1 [16]. However, there remains a gap in the current clinical knowledge about the range of segmental motion in asymptomatic participants and those with a diagnosis of LI which has not yet been studied. This was the reason for developing the new concept of the “sub-threshold level of lumbar instability (STLI)”, defined as the sagittal translation and rotation of each lumbar segment that lies between the asymptomatic or normal range and the range leading to an X-ray diagnosis of LI. The STLI prevalence was reported as 78% amongst participants with CLBP in a previous study that was reported at an international conference on integrative medicine in 2019 at Mae Fah Luang University (Arisa Leungbootnak) [17]. This group of patients with STLI has not been reviewed, although a review is necessary as they are at risk of developing LI, as LI is rarely addressed by conservative treatment. The early detection of patients with STLI may facilitate early appropriate treatment and thereby slow the patient’s progression to LI.

The gold-standard radiograph measurement has some limitations in terms of assessment, including the cost of radiograms, time required for examination and interpretation, and radiation exposure [18,19]. The detection of patients with STLI in the community remains difficult due to the limitations of X-ray assessment. Thus, a screening tool (questionnaire) for LI patients was developed. The signs and symptoms of LI were studied originally in 1982 by Kirkaldy-Willis and Farfan [20]. More recently, a specific lumbar instability screening tool was first reported by Cook et al. (2006), who performed a Delphi study with 168 physical therapists [21] and developed a screening tool based on subjective signs and symptoms. The screening was also associated with clinical lumbar instability, and was used to inform the diagnosis in many studies where the criteria related to LI were of interest [9,21,22,23,24,25]. A Thai version was subsequently created by a group of Thai physical therapists [26], who evaluated its construct validity; in 2020, the same group used the questionnaire as a tool for screening patients with LI among CLBP patients [23]. They found that a score of at least 7/14 correlated with having LI; this score was used as an inclusion criterion for participants with LI among patients with CLBP. The tool was also translated (from English into Brazilian-Portuguese) [22] and tested for reliability [9,21,22,24,25,26].

Questionnaires to evaluate the signs and symptoms of patients with lumbar instability are easy to use in clinical practice [21]; however, to date, no studies have used a specific LI screening tool for patients with STLI in the early STLI stages. The results of this study could be beneficial for the early detection of STLI in patients with CLBP using minimally invasive assessment methods; these patients may be at the pre-stage of LI.

## 2. Materials and Methods

### 2.1. Design and Setting

The study design was cross-sectional in nature. Participant testing was conducted at the Department of Associated Medical Science, Khon Kaen University, Thailand. The study was approved by the Ethics Committee for Human Research (HE582228) approved on 12 September 2018 at Khon Kaen University, Thailand. This study has been registered in Thai Clinical Trials at http://www.thaiclinicaltrials.org/show/TCTR20180920003 (accessed on 20 September 2019).

### 2.2. Participants

Participants with CLBP were recruited via poster and social media announcements. The inclusion criteria were: (1) having intermittent low back pain for at least 12 weeks, (2) participant age range 20 to 60 years, and (3) a pain numeric rating scale (NRS) score between 4 and 7 [27,28,29]. Participants were excluded if they had: (1) a contraindication to radiographic assessment, such as pregnancy, (2) acute fracture, tumor, or infection, (3) a history of serious neurological or psychiatric disease, (4) previous lumbar fusion surgery, or (5) a diagnosis of radiological lumbar instability (LI), spondylolisthesis, or lumbar disc herniation [1,5,30]. The investigators gained informed written consent from the participants prior to assessment.

### 2.3. Sample Size Determination

This is the first study to specifically investigate STLI, so the rate of disease came from the results of our previous study, reported at an international conference on integrative medicine (Arisa Leungbootnak, 7 October 2019) in 2019 at Mae Fah Luang University, Thailand (prevalence of STLI = 78%) [17]. The sample size calculation was conducted based on a significance level (α) of 0.01 and a precision of estimation value (e) of 0.10. According to acceptable sensitivity in the physical therapy field, (P) was set as 80% [31], meaning 135 participants were required.

### 2.4. Instruments

#### 2.4.1. Radiographic Assessment and Measurement

**Figure 1:**
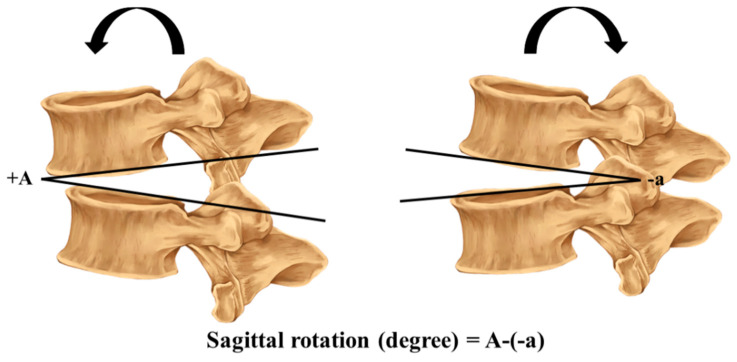
Sagittal rotation: a baseline is drawn passing the anterior and posterior endplate of the upper and lower vertebrae where the difference between the flexion and extension radiograph is measured and calculated.

**Figure 2:**
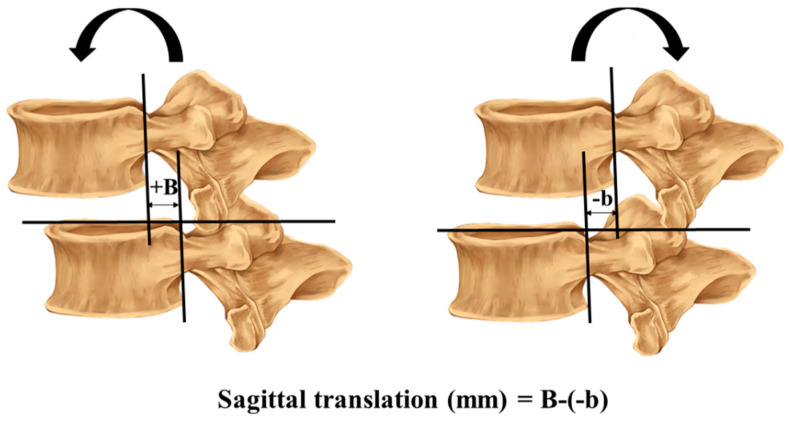
Sagittal translation: a horizontal line is drawn passing the superior endplate of the lower vertebra. Two vertical lines are drawn passing the posterior edge of the upper vertebra and lower vertebra. The distance between the two vertical lines is considered during flexion and extension, and the difference between the two distances from flexion and extension is used in the formula to calculate the segmental translation.

Flexion–extension radiography is the most common form of examination used in studies to provide an imaging diagnosis of lumbar intervertebral instability [32]. The method of measuring the sagittal translation and rotation in the radiographs was previously described by Iguchi [30]. The sagittal translation and rotation were measured during full flexion and extension, and calculated using the formula (sagittal rotation (degrees) = A − (−a) and translation (mm) = B − (−b)), as shown in Figure 1 and Figure 2.

The measurement of sagittal translation and rotation motion of each lumbar segment level from L1 to S1 was compared with ranges set for diagnosing STLI. Values for STLI were defined as: translation range from 1.9 to 4.5 mm at L1/L2, 2.4 to 4.5 mm at L2/L3, 2.7 to 4.5 mm at L3/4, 2.8 to 4.5 mm at L4/L5, and 0.5 to 4.5 mm at L5/S1, or a sagittal plane rotation range from 11.0° to 15° at L1/L2, 12.6° to 15° at L2/L3, 13.3° to 15° at L3/4, 14.7° to 20° at L4/L5, or 12.8° to 25° at L5/S1 [15,16]. The diagnosis of LI required showing either rotation or translation in two segments or rotation and translation in one segment to be classified as STLI [1]. This definition was used to reduce the false-positive rates from the X-ray measurement process [23].

A trained observer (researcher A.L.), who was blinded to other information, measured the translation and rotation of each lumbar segment in all participants using digitalized radiographs from a picture archive and communication (PACS) on a computer at Srinagarind Hospital, Khon Kaen, Thailand. The researcher (A.L.) was trained qualified to assess the radiographs by a radiologist with 30 years of experience. The evaluation practice of A.L.’s X-ray measurement consisted of using the program, pinpointing margins and spinal borders, and drawing the measurement line. The measurement process was practiced until A.L.’s accuracy was rated as satisfactory by the radiologist. Next, A.L. measured each level of the lumbar spine three times. The mean of each of the three measurements was compared with the STLI diagnostic criteria. The intra-rater reliability of X-ray measures was assessed based on the radiographs of 10 participants selected at random. The within researcher ICC (A.L.), measured three times, was 0.998 (95%CI: 0.994–0.999) in translation and 0.994 (95%CI: 0.982–0.998) in rotation. The standard error of measurement (SEM) was 0.025 for translation and 0.058 for rotation. These were a lesser variation than the X-ray measurement [33].

#### 2.4.2. Screening Tool

The specific lumbar instability screening tool had 14 questions and was written in English (Table 1) [23]. This tool was translated and tested for content validity, criteria-related validity, and rater reliability in relation to lumbar instability as reported in previous studies [23,26]. The possible total questionnaire scores ranged from 0 (not correlated with instability) to 14 (strongly correlated with instability). The questions relate to participants’ pain characteristics, positional alteration, muscle spasms, and injury history.

### 2.5. Measurement Procedure

The 135 participants, all diagnosed with CLBP, were split into two groups: those with and without STLI based on X-ray findings. Data were collected from March 2019 through July 2019. The participants were assessed at one visit. On that day, participants signed an informed consent form before starting the study and were evaluated for LBP symptoms. Researcher R.P. asked participants for their demographic information: age, gender, BMI, duration of low back pain, pain scale rating, and smoking history, and then the 14 screening tool questions. Then, participants were evaluated by an orthopedic doctor who ordered an X-ray assessment. X-rays were taken in the full lateral flexion and extension positions, and were performed by a radiologist. The X-ray images were measured by the trained observer (A.L.) after data collection was completed. A.L. was blinded to all other information about the patients.

### 2.6. Statistical Analyses

The data were analyzed and grouped based on participants with and without STLI. Descriptive statistics were used, involving calculations of the mean, standard deviation, and percentage. Significance was set at a *p*-value of less than 0.05 when comparing between groups. Intra-observer reliability was measured using the intraclass correlation coefficient (ICC) for the radiographic measurements.

The receiver operating characteristic (ROC) curve was used to determine the possible cut-off score of lumbar instability screening. Sensitivity, specificity, positive likelihood ratio (LR+), and negative likelihood ratio (LR-) for each cut-off score were calculated. The cut-off score that reached the maximum of sensitivity was taken as the maximum score. The area under the ROC curve (AUG) was used to evaluate the discriminatory ability of the 14 items of the screening tool. Statistical analysis was conducted using STATA ver. 10.0 (StataCorp, College Station, TX, USA).

## 3. Results

### 3.1. Participant Characteristics

Of the 135 participants recruited, 113 participants (83.70%) had STLI. The mean age of all the participants was 35.58 ± 12.02 years. The participants’ gender was 60.74% female. The average age of the participant groups with STLI and without STLI was 35.60 ± 12.46 and 35.45 ± 9.70 years old, respectively. All the continuous data were similar between the groups (*p* > 0.05), as shown in Table 2.

### 3.2. The Screening Tool-Specific STLI Cut-Off Scores

The current study showed that the cut-off score for identifying STLI requires a total of at least six positive responses of a possible score of 14 from the screening tool-specific questions. The sensitivity, specificity, positive likelihood ratio, and negative likelihood ratio were 99.12%, 18.18%, 1.21, and 0.05, respectively, as shown in Table 3. The high sensitivity indicates the potential screening value because the measure rarely misses subjects who have the condition [34]. The area under the ROC curve was 0.73, reflecting sufficient diagnostic accuracy [35].

## 4. Discussion

The impairment or dysfunction of the active, passive, or neural control subsystems in CLBP patients causes LI. Delayed detection of patients with LI can lead to more structural degeneration, which is challenging to improve with conservative treatment. The early-stage detection of LI by physical therapists may help the patient by preventing further structural damage early on; for example, exercise stabilization, which focuses on deep trunk muscle training, can improve or delay the development of lumbar instability [36,37,38]. In the present study, a subjective questionnaire cut-off score for identifying STLI was found to be 6/14.

The Delphi survey results from Cook et al. (2006) listed 14 screening questions to determine the clinical lumbar instability in the CLBP participants [21]. The current study results showed that at least six positive answers to the fourteen listed lumbar instability questions among the CLBP participants led to a diagnosis of STLI. This result was similar to Kumar (2018) and Puntumetakul et al. (2014), who selected 7/13 positive responses to similar screening questions that could act as the criteria for diagnosing lumbar segmental instability [9,24]. However, when compared with the study by Chatprem et al. in 2020, our result was lower than their cutoff score (7/14) [23]. This may be because the participants in this study had less translation and rotation (STLI) than those in their (LI) study.

**Figure 3:**
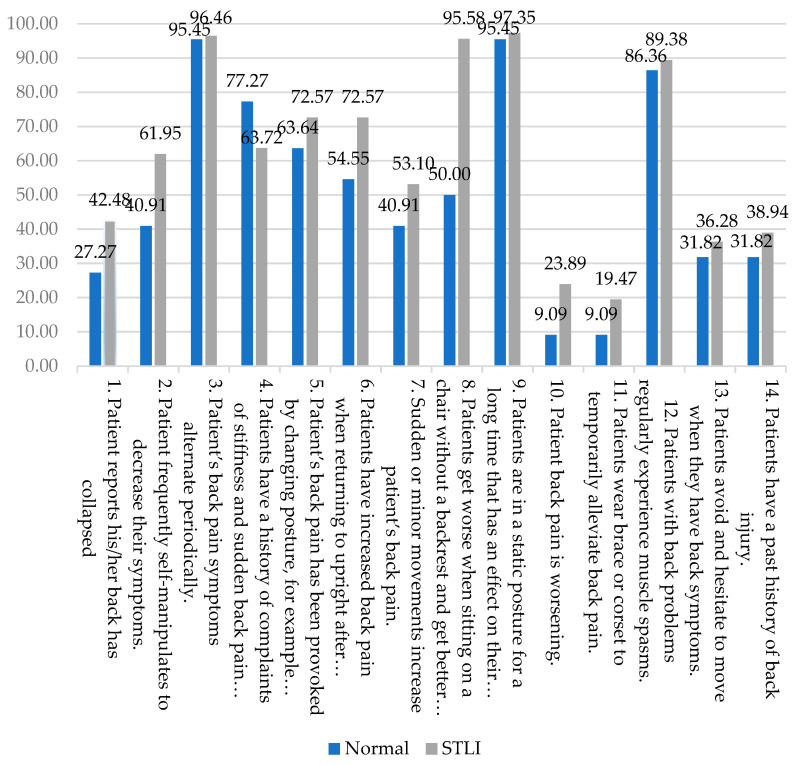
Comparison of the percentage of positive answers to the lumbar instability screening tool questions in chronic low back pain patients with and without the sub-threshold level of lumbar instability.

In the current study, three key symptoms were frequently reported by the participants with CLBP, that led to a positive diagnosis of STLI: (1) back pain alternating occasionally (97.35%); (2) worsening pain when sitting on a chair without a backrest and less pain when sitting on a chair with a backrest (96.46%); and (3) increasing back pain when maintaining prolonged static postures (95.58%) (Figure 3). The symptom of back pain alternating periodically may arise from the spinal unit moving together while the passive subsystem is in fixed alignment. During spinal movement, the passive subsystem is unable to restrain shear forces that arise from muscle contraction and gravity. A possible reason underlying this symptom is that one or more of the spinal subsystems that cause instability may be dysfunctional due to injury or degeneration [6]. In the sitting position, many people experience musculoskeletal discomfort, principally in the lower back and buttock areas, where the discomfort increases significantly during prolonged sitting [39]. The responses in the current study differed from some of those reported in a farming population by Puntumetakul et al. (2014) [9]. The responses in that study consisted of: (1) frequent episodes of muscle spasm (93%); (2) worsening symptoms with sustained postures (90%); and (3) frequent episodes of symptoms (88%). The differences between these two studies included: the participant occupation, number and age of participants, and use of different criteria to determine the presence of lumbar instability (clinical symptoms in the former study but X-ray evaluation in the current study). Nevertheless, the characteristic of worsening symptoms with sustained postures was the same in both studies.

An optimal cut-off point for LI patients of at least 7 of 14 scores was reported by Chatprem et al. in 2020 [23]. The 7/14 score was based on the maximum summation of sensitivity and specificity, which differed by one point from the current study in choosing the highest sensitivity for screening [34]. However, they reported three items as receiving 100% positive responses in LI, and two out of their three fit with the top responses in our study. These symptoms were prolonged sitting and sustained posture. The possible reasons for the lower score in the STLI participants may be twofold. First, the number of lumbar instability participants (STLI, 113 or 83.70%) was higher in the current study than the number of patients with LI in Chatprem’s study. Second, the STLI criteria in the current study required less translation and rotation compared with LI. Less severe LI may lead to a lower score when comparing LI and STLI. However, considering their study in terms of the highest sensitivity for screening, a score of at least 7 of 14 remains the cut-off point.

Our participant demographic characteristics, particularly gender, have no difference between those with chronic low back pain with STLI and without STLI. Therefore, this minimizes other factors that may influence our results. The current study demonstrated that a screening tool specific for LI is beneficial in accurately identifying STLI among CLBP participants. In a clinical setting, choosing the cut-off scores of at least 6/14 positive points can be used to screen patients with CLBP for STLI responses. Interestingly, patients with positive STLI also had positive results on items 3, 8, and 9 of the screening tool questionnaire. Moreover, the effect size was large [40].

This study has useful implications for clinicians; however, there are some limitations. First, the participants experiencing pain while the flexion–extension radiographs were taken may have shown a lower range of instability than they actually had. Second, the sample size was small. Third, our participants had a wide age range, meaning they may have had a varied state of disc degeneration. Fourth, there may have been an overlap with the upper range of normal asymptomatic participants with those in the STLI group. Future studies could consider using a magnetic resonance image assessment to determine the stage of disc degeneration, to help improve the strength of the results.

## 5. Conclusions

The findings of the current study revealed useful information for identifying STLI using a specific screening tool for patients with LI. Although this screening tool is effective for assessing the lumbar spinal instability in an outpatient clinic without the need for sophisticated and expensive equipment, high specificity tests, such as the passive lumbar extension test (PLE), are still recommended to confirm the diagnosis of STLI.

## Figures and Tables

**Table 1:** Lumbar instability screening tool.

Question 14 items	Yes (1)	No (0)
1. Patient reports his/her back has collapsed.		
2. Patient frequently self-manipulates to decrease their symptoms.		
3. Patient’s back pain symptoms alternate periodically.		
4. Patient has a history of complaints of stiffness and sudden back pain when twisting or bending their back.		
5. Patient’s back pain has been provoked by changing posture, for example standing up from sitting, etc.		
6. Patient has increased back pain when returning to upright after forward bending.		
7. Sudden or minor movements increase patient’s back pain.		
8. Patient gets worse when sitting on a chair without a backrest and gets better when sitting on a chair with backrest.		
9. Patient reports being in a static posture for a long time has an effect on their back problem.		
10. Patient’s back pain is worsening.		
11. Patient wears a brace or corset to temporarily alleviate back pain.		
12. Patient with back problems regularly experiences muscle spasms.		
13. Patient avoids or hesitates to move when they have back symptoms.		
14. Patient has a past history of back injury.		
Total score		

**Table 2:** Demographic characteristics of participants.

Variable	Total (*n* = 135)	With STLI (*n* = 113)	Without STLI (*n* = 22)	*p*-Value
Age (years)	35.58 ± 12.02	35.60 ± 12.46	35.45 ± 9.70	0.95
Gender (%)				
Male	53 (39.26)	45 (39.82)	8 (36.36)	0.82
Female	82 (60.74)	68 (60.18)	14 (63.64)	
BMI (kg/m^2^)	22.16 ± 2.10	22.29 ± 2.10	21.48 ± 2.04	0.10
Duration of current symptoms (months)	27.33 ± 27.07	26.40 ± 27.54	32.14 ± 24.57	0.37
NRS (pain)	4.63 ± 0.94	4.57 ± 0.88	4.95 ± 1.21	0.17
Smoking history (%)				
Yes	12 (8.89)	10 (8.85)	2 (9.09)	1.00
No	123 (91.11)	103 (91.15)	20 (90.91)	

Note: STLI: sub-threshold level of lumbar instability; BMI: body mass index; NRS: numeric rating scale.

**Table 3:** The screening tool-specific STLI cut-off scores.

Cut-Off Value	Sensitivity (%)	Specificity (%)	LR+	LR−	AUG (95%CI)
≥5	100.00	0.00	1.00		0.73 (0.61–0.84)
≥6	99.12	18.18	1.21	0.05	
≥7	90.27	31.82	1.32	0.31	
≥8	69.91	68.18	2.20	0.44	
≥9	46.02	72.73	1.69	0.74	
≥10	31.86	95.45	7.01	0.71	
≥11	17.70	100.00		0.82	
≥12	5.31	100.00		0.95	
≥13	2.65	100.00		0.97	
≥14	0.88	100.00		0.99	
14	0.00	100.00		1.00	

## Data Availability

The data will be available for anyone who wishes to access them for any purpose and contact should be made via the Corresponding author rungthiprt@gmail.com.

## References

[1] Fritz J.M., Piva S.R., Childs J.D. (2005). Accuracy of the clinical examination to predict radiographic instability of the lumbar spine. Eur. Spine J..

[2] Panjabi M.M. (1992). The stabilizing system of the spine. Part I. Function, dysfunction, adaptation, and enhancement. J. Spinal Disord..

[3] Panjabi M.M. (2003). Clinical spinal instability and low back pain. J. Electromyogr. Kinesiol..

[4] Last A.R., Hulbert K. (2010). Chronic low back pain: Evaluation and management. S. Afr. Fam. Pract..

[5] Abbott J.H., McCane B., Herbison P., Moginie G., Chapple C., Hogarty T. (2005). Lumbar segmental instability: A criterion-related validity study of manual therapy assessment. BMC Musculoskelet. Disord..

[6] Ahn K., Jhun H.J. (2015). New physical examination tests for lumbar spondylolisthesis and instability: Low midline sill sign and interspinous gap change during lumbar flexion-extension motion Orthopedics and biomechanics. BMC Musculoskelet. Disord..

[7] Hicks G.E., Fritz J.M., Delitto A., McGill S.M. (2005). Preliminary development of a clinical prediction rule for determining which patients with low back pain will respond to a stabilization exercise program. Arch. Phys. Med. Rehabil..

[8] Kasai Y., Morishita K., Kawakita E., Kondo T., Uchida A. (2006). A new evaluation method for lumbar spinal instability: Passive lumbar extension test. Phys. Ther..

[9] Puntumetakul R., Yodchaisarn W., Emasithi A., Keawduangdee P., Chatchawan U., Yamauchi J. (2014). Prevalence and individual risk factors associated with clinical lumbar instability in rice farmers with low back pain. Patient Prefer. Adherence.

[10] Vanti C., Conti C., Faresin F., Ferrari S., Piccarreta R. (2016). The Relationship Between Clinical Instability and Endurance Tests, Pain, and Disability in Nonspecific Low Back Pain. J. Manip. Physiol. Ther..

[11] Kotilainen E., Valtonen S. (1993). Acta Neurochirurgica Clinical Instability of the Lumbar Spine After Mierodiseectomy. Acta Neurochir..

[12] Tang S., Qian X., Zhang Y., Liu Y. (2016). Treating low back pain resulted from lumbar degenerative instability using Chinese Tuina combined with core stability exercises: A randomized controlled trial. Complementary Ther. Med..

[13] Panjabi M. (1992). The stabilizing system of the spine. Part II. Neutral zone and instability hypothesis. J. Spinal Disord..

[14] Dang L., Zhu J., Liu Z., Liu X., Jiang L., Wei F., Song C. (2020). A new approach to the treatment of spinal instability: Fusion or structural reinforcement without surgery?. Med. Hypotheses.

[15] White M.M., Panjabi A. (1990). Clinical Biomechanics of the Spine.

[16] Staub B.N., Holman P.J., Reitman C.A., Hipp J. (2015). Sagittal plane lumbar intervertebral motion during seated flexion-extension radiographs of 658 asymptomatic nondegenerated levels. J. Neurosurg. Spine.

[17] Leungbootnak A. The range of lumbar spine motion in Thai patients with non-radiological lumber instability (pilot study). Proceedings of the 1st International Conference on Integrative Medicine.

[18] Alqarni A.M., Schneiders A.G., Hendrick P.A. (2011). Clinical tests to diagnose lumbar segmental instability:A systematic review. J. Orthop. Sports Phys. Ther..

[19] Ozcete E., Boydak B., Ersel M., Kiyan S., Uz I., Cevrim O. (2015). Comparison of conventional radiography and digital computerized radiography in patients presenting to emergency department. Turk. J. Emerg. Med..

[20] Kirkaldy-Willis W.H., Farfan H. (1994). Instability of the lumbar spine. Clin. Orthop. Relat. Res..

[21] Cook C., Brismée J.M., Sizer P.S. (2006). Subjective and objective descriptors of clinical lumbar spine instability: A Delphi study. Man. Ther..

[22] Araujo A.C., Da Cunha Menezes Costa L., De Oliveira C.B.S., Morelhão P.K., De Faria Negrão Filho R., Pinto R.Z., Costa L.O.P. (2017). Measurement Properties of the Brazilian-Portuguese Version of the Lumbar Spine Instability Questionnaire. Spine.

[23] Chatprem T., Puntumetakul R., Boucaut R., Wanpen S., Chatchawan U. (2020). A Screening Tool for Patients With Lumbar Instability: A Criteria-related Validity of Thai Version. Spine.

[24] Kumar S.P. (2011). Efficacy of segmental stabilization exercise for lumbar segmental instability in patients with mechanical low back pain: A randomized placebo controlled crossover study. N. Am. J. Med. Sci..

[25] Saragiotto B.T., Maher C.G., New C.H., Catley M., Hancock M.J., Cook C.E., Hodges P.W. (2018). Clinimetric testing of the Lumbar Spine instability questionnaire. J. Orthop. Sports Phys. Ther..

[26] Chatprem T., Puntumetakul R., Yodchaisarn W., Siritaratiwat W., Boucaut R., Sae-jung S. (2020). A Screening Tool for Patients With Lumbar Instability: A Content Validity and Rater Reliability of Thai Version. J. Manip. Physiol. Ther..

[27] Wennberg P., Möller M., Sarenmalm E.K., Herlitz J. (2020). Evaluation of the intensity and management of pain before arrival in hospital among patients with suspected hip fractures. Int. Emerg. Nurs..

[28] Fejer R., Jordan A., Hartvigsen J. (2005). Categorising the severity of neck pain: Establishment of cut-points for use in clinical and epidemiological research. Pain.

[29] Hanley M.A., Masedo A., Jensen M.P., Cardenas D., Turner J.A. (2006). Pain interference in persons with spinal cord injury: Slassification of mild, moderate, and severe pain. J. Pain.

[30] Maigne J.Y., Lapeyre E., Morvan G., Chatellier G. (2003). Pain immediately upon sitting down and relieved by standing up is often associated with radiologic lumbar instability or marked anterior loss of disc space. Spine.

[31] Van der Wulf P., Meyne W., Hagmeijer R.H.M. (2000). Clinical tests of the sacroiliac joint. A systematic methodological review. Part 2: Validity. Man. Ther..

[32] Leone A., Guglielmi G., Cassar-Pullicino V.N., Bonomo L. (2007). Lumbar intervertebral instability: A review. Radiology.

[33] Saiklang P., Puntumetakul R., Swangnetr Neubert M., Boucaut R. (2019). Effect of time of day on height loss response variability in asymptomatic participants on two consecutive days. Ergonomics.

[34] Goetzinger K.R., Tuuli M.G., Odibo A.O. (2011). Statistical analysis and interpretation of prenatal diagnostic imaging studies, part 3: Approach to study design. J. Ultrasound Med..

[35] Simundic A.M. (2009). Measures of Diagnostic Accuracy: Basic Definitions. Ejifcc.

[36] Puntumetakul R., Areeudomwong P., Emasithi A., Yamauchi J. (2013). Effect of 10-week core stabilization exercise training and detraining on pain-related outcomes in patients with clinical lumbar instability. Patient Prefer. Adherence.

[37] Puntumetakul R., Saiklang P., Tapanya W., Chatprem T., Kanpittaya J., Arayawichanon P., Boucaut R. (2021). The effects of core stabilization exercise with the abdominal drawing-in maneuver technique versus general strengthening exercise on lumbar segmental motion in patients with clinical lumbar instability: A randomized controlled trial with 12-month follow-up. Int. J. Environ. Res. Public Health.

[38] Javadian Y., Akbari M., Talebi G., Taghipour-Darzi M., Janmohammadi N. (2015). Influence of core stability exercise on lumbar vertebral instability in patients presented with chronic low back pain: A randomized clinical trial. Casp. J. Intern. Med..

[39] Søndergaard K.H.E., Olesen C.G., Søndergaard E.K., de Zee M., Madeleine P. (2010). The variability and complexity of sitting postural control are associated with discomfort. J. Biomech..

[40] Salgado J.F. (2018). Transforming the area under the normal curve (AUC) into cohen’s *d*, pearson’s *r_pb_*, odds-ratio, and natural log odds-ratio: Two conversion tables. Eur. J. Psychol. Appl. Leg. Context.

